# Investigation of the Crystallographic Evolution Sequence of Nano-Precipitation via HRTEM in Cu-Bearing Ultra-Low Carbon Steel

**DOI:** 10.3390/nano14161335

**Published:** 2024-08-10

**Authors:** Weina Zhang, Zhanjie Gao, Hao Wei, Huimin Zhang, Zejin Chen, Wenying Xue, Yongfeng Shen, Zhenyu Liu

**Affiliations:** 1 State Key Laboratory of Rolling and Automation, Northeastern University, Shenyang 110819, China; 2 School of Materials Science and Engineering, Northeastern University, Shenyang 110819, China; shenyf@smm.neu.edu.cn

**Keywords:** Cu-bearing ultra-low carbon steel, HRTEM, 3R structure, 9R structure, evolution sequence

## Abstract

The precipitation behavior of Cu-bearing ultra-low carbon steel after step quenching and tempering at 923 K for 0.5–2.5 h was investigated. The size, quantity, and characteristic distribution of nano-precipitates were analyzed using transmission electron microscopy, and the microstructure of B2 (an ordered structure belonging to the body-centered cubic structure), 9R (a special triclinic lattice that has characteristics of rhombohedral structure), 3R (a special triclinic lattice like 9R), and FCT (face-centered tetragonal lattices) were accurately determined. The relationship between nano-precipitates and mechanical properties under different heat treatment processes was obtained, revealing that nano-precipitates effectively enhanced the yield strength of Cu-bearing ultra-low carbon steel. There were two forms of crystal structure evolution sequence of precipitation: B2→multi twin 9R→detwined 9R→FCT→FCC and B2→multi-twin 9R→detwinned 9R→3R→FCT→FCC. The morphology of the precipitated particles during the growth process changed from spherical to ellipsoidal and finally to rod-shaped. It was proven that a stable 3R structure existed due to the coexistence of 9R, 3R, and FCT structures in the same precipitate particle.

## 1. Introduction

Cu-bearing ultra-low carbon steels, which require high strength, good low temperature toughness, and excellent weldability, have drawn much attention for the construction of hull structures, offshore drilling platforms, oil pipelines, and bridges [1,2,3,4]. In Cu-bearing high-strength low-alloy (HSLA) steels (e.g., HSLA-80, HSLA-100, and NUCu-140), the carbon content is kept below 0.08 wt.% and 1–2 wt.% Cu is added to compensate for the reduction in strength due to lower C concentration [5,6,7,8]. The addition of Cu can significantly improve the strength through precipitation strengthening, which has received much attention in recent years [9,10,11]. Therefore, understanding the Cu precipitation behavior and the evolution of precipitation mechanisms is crucial for improving the properties of Cu-bearing steels.

Many research papers have been published on the evolution of the precipitation morphology [12,13], crystal structure [14,15,16], and chemical composition [17,18,19,20,21] of Cu particles [12,13,14,15,22]. Han et al. [16,23,24] reported a B2 ordered structure or cluster with an average radius of 2 nm. As the ordered structure or cluster grew, the precipitate lost coherence and transformed into a twinned monoclinic martensite 9R phase with a radius of 3.5–7.5 nm [25,26]. A semicoherent ellipsoidal 9R structure and an orthorhombic 3R structure were found at a radius range of 9 to 15 nm [27,28]. Fully incoherent ε-FCC copper precipitates were formed at a radius larger than 20 nm [29,30].

It is generally accepted that the crystal structure evolution of Cu precipitates may follow the sequence of “body-centered cubic (BCC)→9R→3R→face-centered cubic (FCC)” [31,32,33,34,35]. Heng et al. [14,36] reported that 9R Cu can directly transform into a twin FCC structure, bypassing the intermediate transition stage from 9R Cu to 3R Cu. Othen et al. [25] proposed that the 9R Cu structure was first detwinned and then transformed into a 3R Cu structure. Monzen [33] reported that 9R Cu first transformed to FCT Cu and then to FCC Cu. Recent studies have mainly focused on the structure and evolution sequence of Cu, but controversy persists due to the lack of direct crystallographic observations on the intermediate transformation process of 9R→FCC.

In recent years, it has been recognized that a multiphase organization consisting of “soft” ferrite/pearlite and “hard” bainite/martensite phases can improve the impact properties of Cu-bearing ultra-low carbon steels. The phase transformation after quenching results in a full solid solution of the alloying elements and exhibits specific laminations, laths, and a high density of dislocations or twins. Tempered bainite, with its inherent combination of high strength and toughness, significantly improves the mechanical properties of steel, but the brittleness of tempered bainite decreases its plasticity and impact toughness. Therefore, to obtain excellent overall mechanical properties, a step-quenching heat treatment process (SQT) was introduced in the QT process to provide a significant combination of strength and toughness of steel [37,38]. The SQT process means that a multiphase organization of ferrite, pearlite, and martensite can be obtained by austenitizing, cooling directly to the critical temperature range, and then cooling rapidly without a reheating process [39,40]. Compared to the intercritical heat treatment process (IQT), energy and time are saved. Therefore, it is necessary to study the effect of the heat treatment process on the microscopic structure of ultra-low-carbon Cu-bearing steels. 

In the present work, the crystal structures of Cu precipitates were systematically observed using HRTEM, and the mechanism for crystallographic evolution was established. The precipitation behavior of a Cu-bearing ultra-low carbon steel after a step-quenching heat treatment process was analyzed. This study is conducive to deepening our comprehension of the precipitation behavior and evolution sequence in Cu-bearing ultra-low carbon.

## 2. Materials and Methods

**Figure 1:**
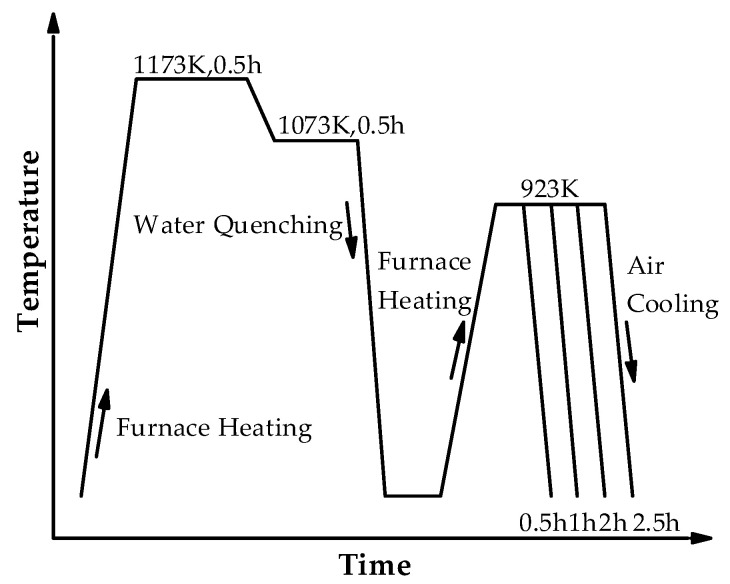
Step quenching and tempering (SQT) heat treatment process.

The composition of the investigated Cu-bearing ultra-low carbon steel was as follows: 0.02 C, 1.52 Cu, 1.54 Ni, 0.58 Cr, 0.33 Si, 0.29 Mo, 0.03 V, and 0.05 Al (wt.%, bal. Fe). The investigated steel was melted by a vacuum induction furnace under an argon atmosphere, followed by forging and cutting into billets with dimensions of 80 mm × 80 mm × 120 mm. The billets were austenitized at 1473 K for 2 h and then hot rolled to a final thickness of 12 mm using a 450 mm mill (State Key Laboratory of Rolling and Automation, Shenyang, China). Then, the steel plate was subjected to a step quenching + tempering (SQT) heat treatment process, as shown in Figure 1. The samples were placed in a chamber furnace at 1173 K and held for 0.5 h to ensure that the steel was fully austenitized and homogenized. Then, the temperature was reduced to 1073 at 10 °C/min and held for 0.5 h, followed by quenching. To obtain a large number of nano-phases, the samples continued to be tempered at 923 K. The tempering times were 0.5, 1, 2, and 2.5 h, followed by cooling to room temperature. Only one quenching process was used to cool down the fully austenitized steel plate directly to the inter-critical temperature, which eliminated the need for reheating, saved heating costs, shortened the process, and improved production efficiency.

The microstructural examination was conducted using field emission scanning electron microscopy (Zeiss, ULTRA55, Oberkochen, Germany). These samples were mechanically polished and then etched in 2% nital solution for about 15–30 s. Transmission electron microscopy (TEM) and high-resolution TEM analyses were examined in a FEI Tecnai G^2^ F20 TEM ( FEI Company, OREGON, USA). Thin foils were prepared using sandpapers. These foils were then electropolished in an electrolyte mixture containing 92% ethyl alcohol and 8% perchloric acid. Tensile samples were cut from the plates along the rolling direction (RD). Longitudinal round tensile specimens of 6 mm diameter and 30 mm gauge length were machined. Tensile testing was conducted in a CMT-5105 testing machine with a tensile rate of 1 mm/min.

## 3. Results and Discussion

### 3.1. Microstructure Characterization

**Figure 2:**
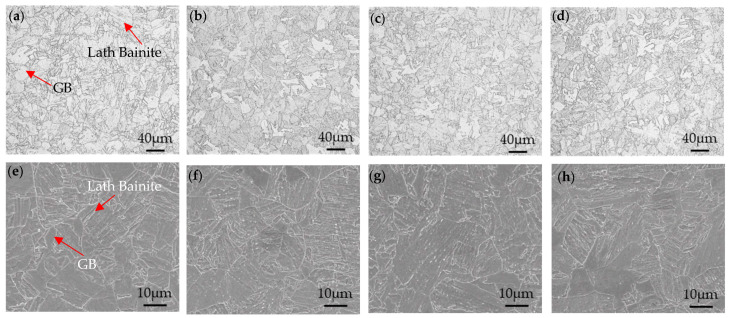
OM and SEM images of the microstructure under different heat treatment conditions: (**a**,**e**) 0.5 h, (**b**,**f**) 1 h, (**c**,**g**) 2 h, and (**d**,**h**) 2.5 h.

Figure 2 shows the optical microstructure and SEM image of the steel after quenching and tempering treatment at stepped temperatures. It can be seen that the steel was mainly composed of tempered bainite. This heating process fully austenitized the steel and reduced the internal stresses caused by quenching. With the extension of the tempering time, the bainite was gradually transformed into tempered bainite. It could be observed that bainite recrystallization occurred, and the number of laths of bainite decreased significantly as the tempering time increased.

**Figure 3:**
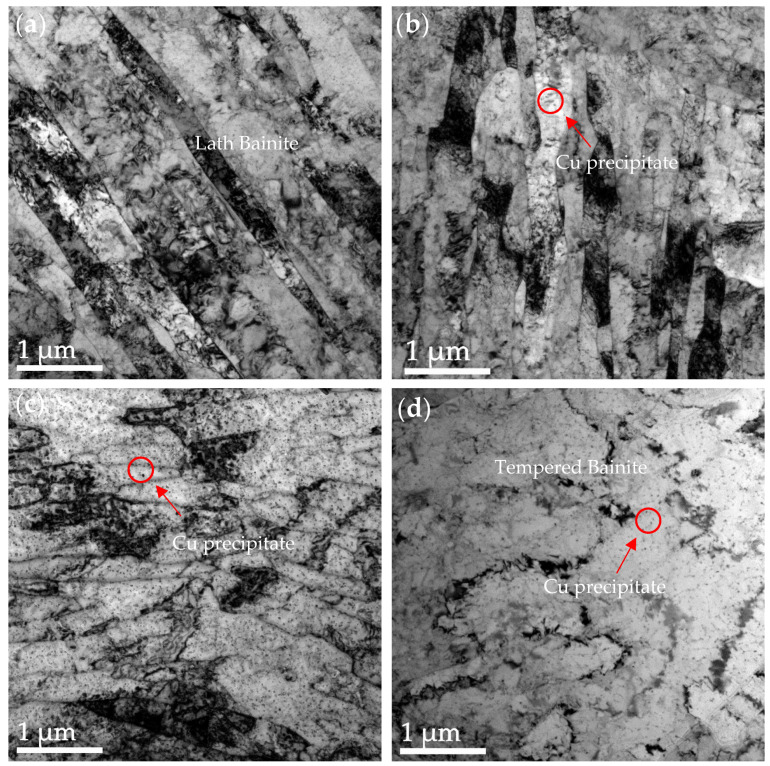
TEM images of the microstructure under different heat treatment conditions: (**a**) 0.5 h, (**b**) 1 h, (**c**) 2 h, and (**d**) 2.5 h.

Figure 3 shows the TEM morphology of the steel under different heat treatment conditions. At the initial 0.5 h of tempering, the obtained tempered bainite was mainly lath. During the SQT process, when quenching at 1073 K, the bainite formed had a low carbon content because the solubility of carbon and alloying elements in austenite was lower than that at 1173 K. The lower carbon bainite had a finer and more uniform distribution of carbides, which helped to achieve a better balance between strength and toughness. As the tempering time increased to 1 h, the tempered bainite underwent a significant restitution process, while the amount of copper precipitates increased. The precipitates initially appeared as fine particles and increased with the tempering time. The precipitates within the grain boundaries or bainite matrix helped to reduce lattice distortion and enhanced the ductility and toughness of the material. When the tempering time was extended to 2 h, the tempered bainite underwent restitution and recrystallization. Tempering allowed these defects to be reorganized or eliminated through slip and climb mechanisms, and dislocation interactions similarly led to a decrease in the number of dislocations, which in turn reduced lattice distortions and released internal stresses. During prolonged tempering, the bainite formed equiaxed grains at a tempering time of 2.5 h.

**Figure 4:**
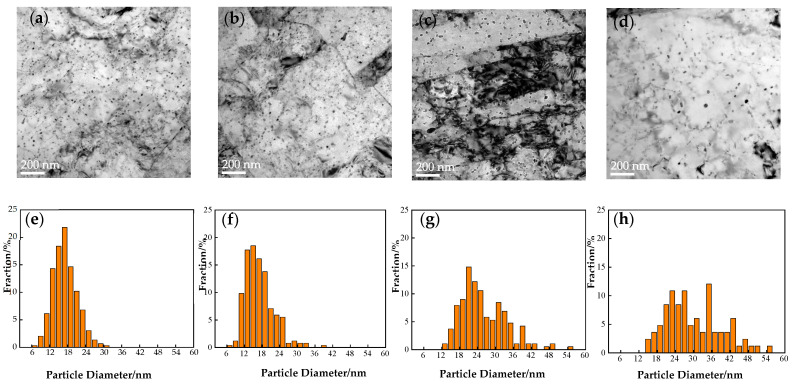
Statistical analysis of precipitation particle size under different heat treatment conditions: (**a**,**e**) 0.5 h, (**b**,**f**) 1 h, (**c**,**g**) 2 h, and (**d**,**h**) 2.5 h.

Figure 4 shows the TEM morphology and precipitated particle size statistics of the nano-phase of the steel under different tempering processes. The average size and number density statistics of the precipitated particles are shown in Table 1. We took approximately 10–15 images of each sample by TEM. We marked the precipitates using the PS software (Photoshop 2018) and calculated the number of precipitates and image area using the Image-Pro Plus software. The image obtained by TEM was a projection of a certain thickness in a three-dimensional space. The thickness of the measurement area was about 50–80 nm, which was an experience value, and we took the middle value of 65 nm for calculation. In this way, the density of the number of precipitated particles could be calculated. As the tempering time increased, the average size increased from 18.5 ± 3.18 nm to 29.2 ± 5.38 nm, and the number density decreased from (2.08 ± 0.5) × 10^21^ m^−3^ to (8.36 ± 2.04) × 10^20^ m^−3^. When the tempering time was 0.5–1 h, the size of the precipitated Cu particles increased slightly with the extension of the tempering time, the number density decreased, and the overall change was not obvious. After quenching, the Cu atoms in the material were in a supersaturated state, and with the start of the tempering treatment, the Cu atoms began to precipitate into nuclei. At a tempering time of 0.5 h, precipitated particles were in the nucleation stage. When the tempering time reached 2 h, the size of Cu precipitation particles increased significantly, and the density decreased sharply. With the extension of the tempering time, Cu atoms in the matrix accelerated the diffusion rate, promoting the growth of nucleated particles and resulting in a significant increase in particle size. When the tempering time was in the range of 2–2.5 h, the size of the precipitated Cu particles increased slowly, and the number density decreased slowly, which was not obvious. With a further increase in tempering time, the available diffusion distance of Cu atoms in the matrix became smaller, leading to diffusion limitation and slowing down of the particle growth rate. Prolonged tempering resulted in the system gradually approaching its thermodynamic equilibrium state, and the kinetics of precipitated Cu particle growth and new nucleation were constrained by the equilibrium, resulting in stabilization of the size and number density.

### 3.2. HRTEM Observations

**Figure 5:**
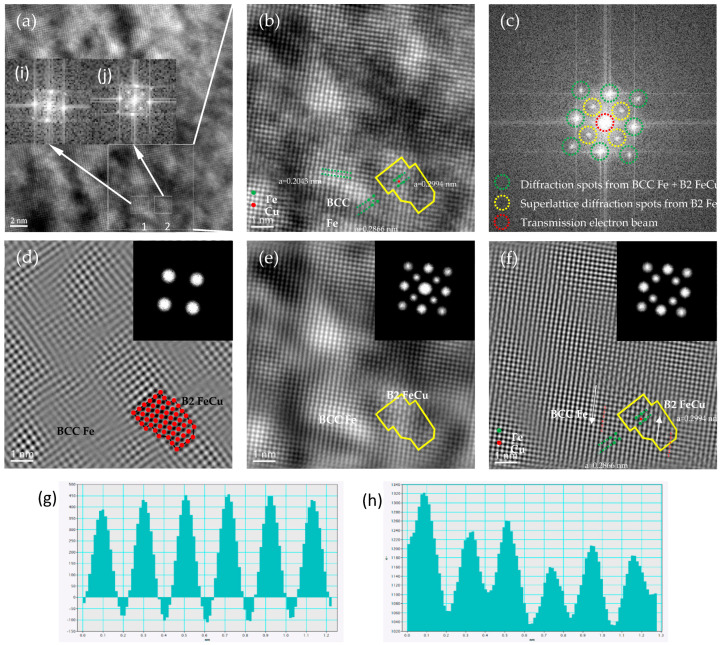
[001]_α_ HRTEM image of the B2 nano-precipitates at 923 K for 0.5 h. (**a**) [001]_α_ HAADF image showing coherent B2 precipitates with a weak bright contrast. (**b**) Enlarged view of the white area of (**a**). (**c**) FFT of (**b**). (**d**) IFFT of (**b**) with 4 diffraction spots. (**e**) IFFT of (**b**) with 13 diffraction spots. (**f**) IFFT of (**b**) with 12 diffraction spots. (**g**) Atomic spacing measurement plot of α-BCC. (**h**) Atomic spacing measurement plot of B2. (**i**) FFT pattern from the α-BCC region. (**j**) FFT pattern from the B2 ordered region.

In the initial stage of precipitation, the B2 ordered phase was first formed, as shown in the matrix in Figure 5. Figure 5a is a high-resolution image of B2 along the [001] _α_ direction, and Figure 5b is a magnified image of the white region in Figure 5a. The interface between the B2 nano-phase (~2 nm) and the α-Fe matrix is indicated by the yellow line (shown in Figure 5b). Figure 5c shows the FFT image of Figure 5b, where the superlattice diffraction spots from the B2 precipitates appeared. The calibration showed that there was an orientation relationship between B2 and the substrate (110)_B2_//(110)_α_, [001]_B2_//[001]_α_. Figure 5d is the IFFT image of Figure 5b, obtained using only the four superlattice diffraction points of B2 precipitation. In Figure 5d, the region containing bright spots is the B2 precipitation, and the interface between the B2 precipitates and the α-Fe substrate can be determined by the bright red spots that have been colored according to the intensity of the brightness. Figure 5e is a denoised image of Figure 5b, which is an IFFT map obtained from thirteen spots containing the transmitted electron beam, including four superlattice diffraction spots from B2 and eight diffraction spots from α-BCC. Figure 5f is the IFFT image of Figure 5b with four superlattice diffraction spots and eight α-BCC diffraction spots selected to compose the image. In the 2D high-resolution image of the α-BCC substrate in the absence of the transmitted beam, the spots show a uniform brightness. Figure 5f illustrates the possible arrangement of Fe and Cu atoms of B2 within α-BCC, where green indicates Fe atoms and red represents Cu atoms. The spacing of the B2 nano-ordered phase at the (1 1 0) B2 crystal plane was 0.2043 nm, and their lattice constant was 0.2994 nm (0.2887 [41] and 0.2900 [42] nm), which was slightly higher than the lattice constant of 0.2866 nm for BCC Fe. The mismatch between α-BCC and B2 was only 4.47% (less than 5%), suggesting that there was a co-lattice relationship between them.

**Figure 6:**
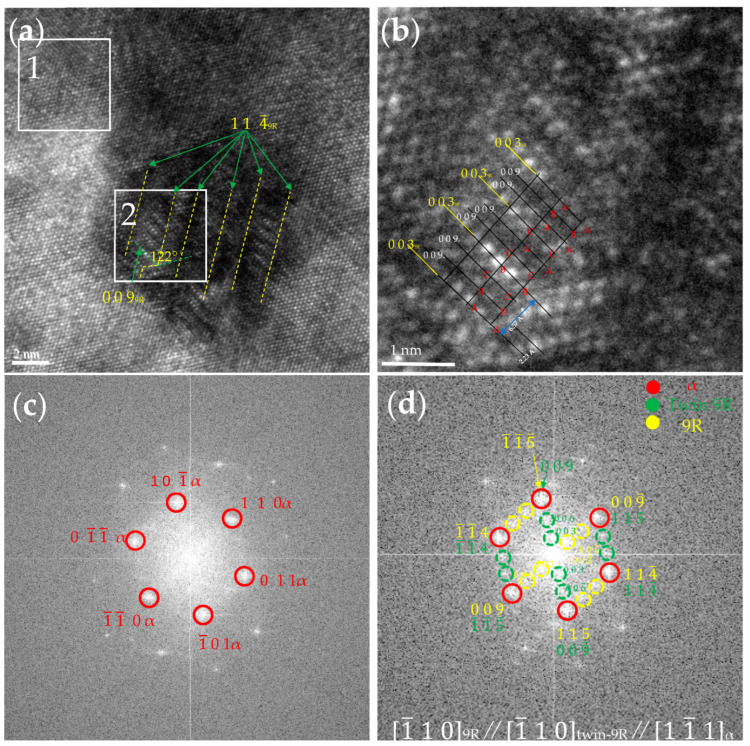
HRTEM image of the 9R structure at 923 K for 0.5 h. (**a**) 9R structure with multi-twins. (**b**) Enlarged view of (**a**). (**c**) FFT of white zone 1 in (**a**) showing the $\left[1\bar{1}1\right]$_α_ axis. (**d**) FFT of white zone 2 in (**a**) showing the $[\bar{1}10$]_9R_ axis.

As the size of the precipitated phase increased, the precipitated structure underwent a change from a B2 ordered to a multi-twinned 9R structure. Figure 6 shows the multi-twinned 9R Cu precipitation phase observed by high-resolution imaging in the $\left[1\bar{1}1\right]$ direction along the α-BCC matrix of the steel after tempering at 923 K for 0.5 h. The length of the phase was about 11.8 nm, and the width was about 9.2 nm. The (1 0 0)_9R_ plane, which is labeled by a dashed line in yellow in Figure 6a, formed an angle with the two adjacent (0 0 9)_9R_ planes of about 122°. A layer of atomic planes with higher phase contrast appeared for every two closely packed atomic planes, a phenomenon attributed to the periodic stacking fault of the (0 0 3) _9R_, (0 0 6) _9R_, and (0 0 9) _9R_ planes. Figure 6b exhibits an enlarged view of region 2 in Figure 6a, where the stacking sequence of 9R structures follows (ABC/BCA/CAB/A). The angle between the (0 0 9)_9R_ plane and the closely stacked direction reached 90°, indicating that this 9R Cu belonged to the orthorhombic crystal system. The (0 0 9)_9R_ crystallographic surface (black line), with an atomic surface spacing of about 0.223 nm, and the (0 0 3)_9R_ crystallographic surface (yellow line), with an atomic surface spacing of about 0.657 nm, which is equivalent to three times the spacing of the (0 0 9)_9R_ close-packed surfaces, agreed with previous studies [30]. Figure 6c,d show the FFT of region 1 and region 2 in Figure 6a. After calibration, it was found that region 1 was the α-BCC matrix. Region 2 consisted of 9R Cu and 9R twin Cu, with red, yellow, and green dashed lines representing the diffraction patterns of BCC Fe, 9R Cu, and twin 9R Cu structures in sequence. The orientation relationship between 9R Cu and the α-BCC matrix was $\left(11\bar{4}\right)$_9R_//(011)_α_, $\left[\bar{1}10\right]$_9R_//$\left[1\bar{1}1\right]$_α_.

**Figure 7:**
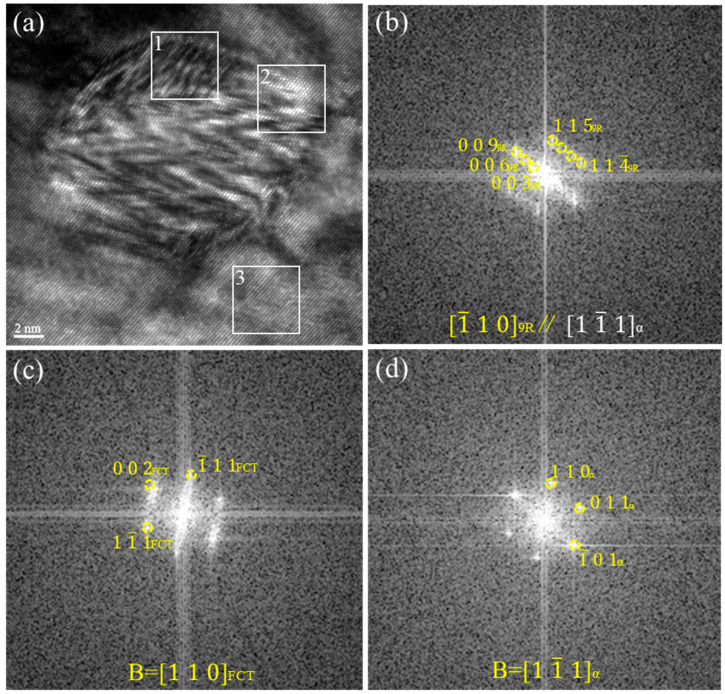
High-resolution image analysis of the steel at 923 K for 0.5 h. (**a**) HRTEM image of 9R and FCT Cu. (**b**) FFT of white zone 1 in (**a**) showing the $\left[\bar{1}10\right]$_9R_ axis. (**c**) FFT of white zone 2 in (**a**) showing the [110]_FCT_ axis. (**d**) FFT of white zone 3 in (**a**) showing the $\left[1\bar{1}1\right]$_α_ axis.

Figure 7 shows high-resolution images of the steel along the matrix [1 −1 1]_α_ after tempering at 923 K for 0.5 h. The nano-phase, shown in Figure 7a, was elliptical with a long axis of about 18.28 nm and a short axis of about 14.39 nm. Figure 7b–d are the FFT images of regions 1, 2, and 3 in Figure 7a, respectively. The calibration results showed that the region 1 structure was a 9R Cu structure, the region 2 structure was an FCT structure, and the region 3 structure was a BCC matrix. Regarding the FCT structure, it was found from the calibration that $d_{\left(1\bar{1}1\right)}$ = 0.215 nm, d _(0 0 2)_ = 0.184 nm, the angle between $\left(\bar{1}11\right)$_FCT_ and $\left(1\bar{1}1\right)$_FCT_ was about 70°, and the angle between $\left(\bar{1}11\right)$_FCT_ and (002) _FCT_ was about 55°. The lattice constants a = b = 0.375 nm and c = 0.368 nm were calculated based on the interplanar spacing equation. Figure 7d shows the FFT image of region 3 in Figure 7a and the calibration results. It was observed by HRTEM that the core regions of the nano-precipitation phase exhibited distinctive “V”-shaped Mohr stripes. The formation of these regions was attributed to lattice distortion during the transition from the 9R Cu structure to the FCT structure. Within a single nano-precipitated phase, the de-twinned 9R Cu and FCT Cu structures could coexist. The appearance of V-shaped Moiré fringes proved that the transition from 9R structure to FCT structure occurred synchronously, and the core region occurred before the edge region, mainly due to the total energy of different regions in the nano-phase.

**Figure 8:**
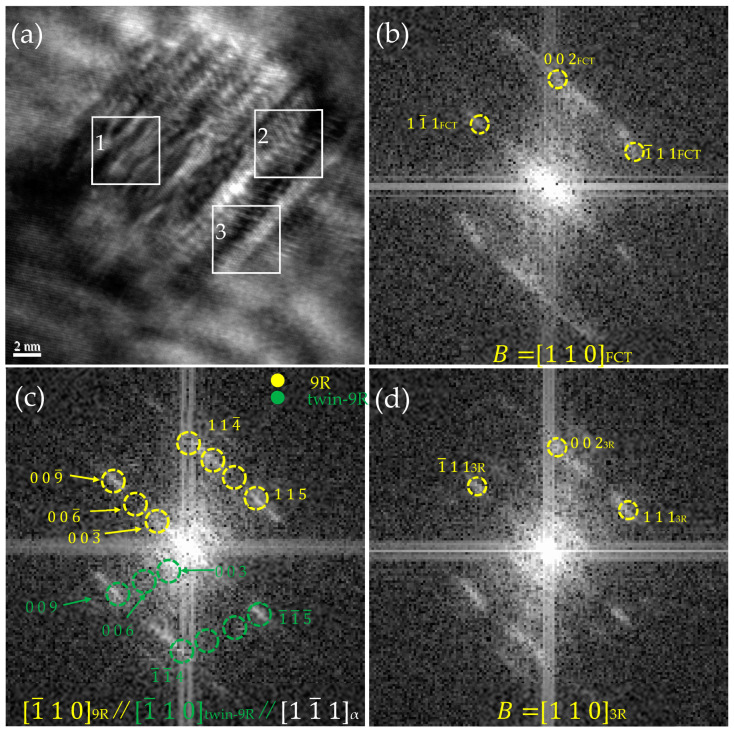
High-resolution image analysis of nano-precipitation at 923 K for 0.5 h. (**a**) HRTEM image of 9R, 3R, and FCT Cu. (**b**) FFT of white zone 2 in (**a**) showing the [110]_FCT_ axis. (**c**) FFT of white zone 1 in (**a**) showing the $[\bar{1}10$]_9R_, $[\bar{1}10$]_twin-9R_ and $[1\bar{1}1$]_α_ axes. (**d**) FFT of white zone 3 in (**a**) showing the [110]_3R_ axis.

Figure 8 shows the HRTEM nano-precipitation images of the steel taken along [1$\bar{1}$1] _α_ direction when tempering at 923 K for 0.5 h. The precipitated particles were characterized by lengths of about 18.9 nm and widths of about 18.5 nm. By Fourier transforming the white regions 1, 2, and 3 in Figure 8a, the diffraction spot calibration showed that region 1 was FCT Cu (as in Figure 8b), region 2 was twin 9R (as in Figure 8c), and region 3 was 3R (as shown in Figure 8d). It can be seen that in a nano-precipitated particle, 9R, 3R Cu, and FCT Cu existed. 3R Cu calculations showed that d _(1 1 1)_ = 0.204 nm and d _(0 0 2)_ = 0.176 nm. The images also directly proved that the nano-precipitated phase transition was from 9R to 3R and finally to the crystallographic evolution of FCT Cu. The 9R Cu twin boundaries of the nano-deposited particles shifted to form 3R Cu and FCT Cu by eliminating the stacking fault. There was also direct evidence of the crystallographic evolution of Cu precipitation.

### 3.3. Mechanical Properties

**Figure 9:**
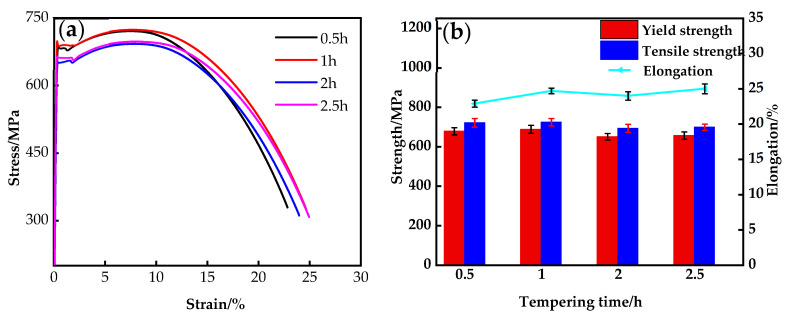
Tensile properties of the steel under different heat treatment conditions: (**a**) typical stretch curve; (**b**) tensile properties change trend.

Figure 9 shows the stress–strain curves (Figure 9a) and the trend of property changes (Figure 9b) of the steel under different tempering times. The yield platform appeared on the tensile curves of the steel at different tempering times, which was caused by movable dislocations during the heat treatment process. Under the SQT process, with the extension of the tempering time, the overall changes were smooth, the strength gradually increased and then decreased before maintaining stability, and the elongation was also affected, as shown in Figure 9b. The yield strength, tensile strength, and elongation of the steel with different tempering times are shown in Table 2. This showed that with the extension of the tempering time, from 0.5 to 1 h, the redistribution of Cu through solid solution strengthening increased the material strength and compensated for the decrease in the contribution of precipitation strengthening. The equilibrium between the precipitation strengthening effect and the tempering softening effect was reached when the tempering time was between 2 and 2.5 h. SQT treatment provided a wider range of tempering time, and excellent mechanical properties could be obtained from a tempering time of 0.5–2.5 h. SQT treatment can be used to improve the strength and elongation of the material and to improve the strength of the material by solution strengthening. This process ensures that the properties of the material are maintained while at the same time significantly reducing energy and time costs, thus increasing productivity and economic efficiency.

**Figure 10:**
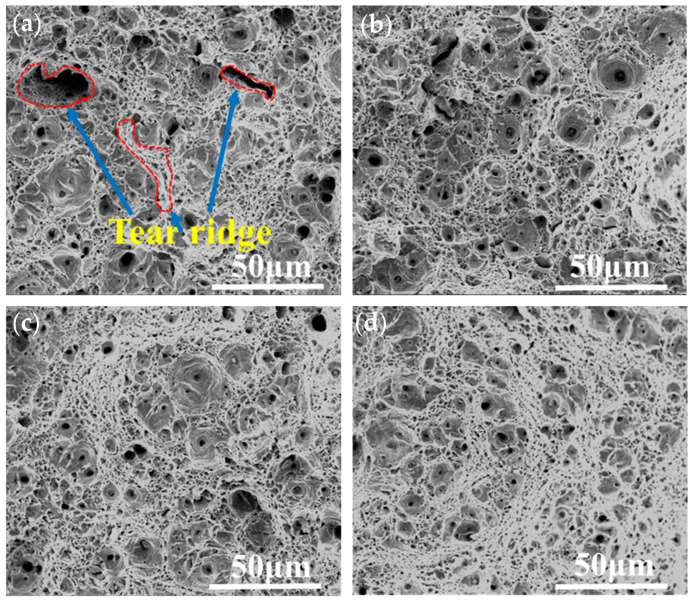
Room temperature tensile fracture morphology under different heat treatment conditions: (**a**) 0.5 h, (**b**) 1 h, (**c**) 2 h, and (**d**) 2.5 h.

Figure 10 shows the tensile fracture morphology at different tempering times. From the figure, it can be seen that the tensile fracture mode was a ductile fracture. In the early stage of tempering (0.5–1 h), the microstructure of the steel began to recover, and the instability of the bainite decreased, forming smaller toughness dimples and exhibiting higher toughness on the fracture surface. The smaller the size and depth of the tear ridge, the better the material’s toughness. When the tempering time was between 2 and 2.5 h, the tearing ridge almost disappeared. As the size of the nano-phase increased and the quantity density decreased, larger precipitations reduced the total area of the interface with the matrix phase, thereby alleviating the formation of cracks in some cases.

### 3.4. The Crystallographic Evolution Sequence

**Figure 11:**
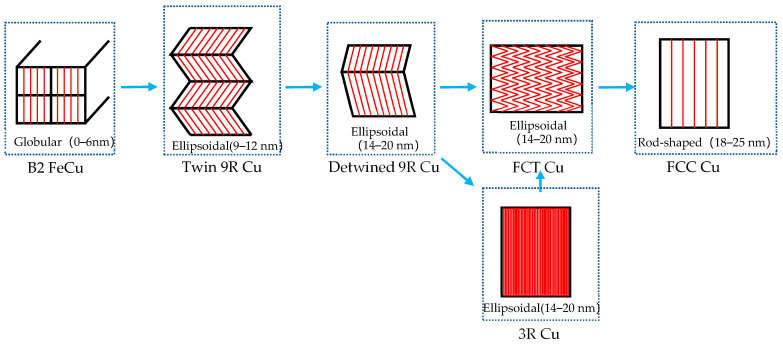
Schematic of the evolution sequence of Cu precipitation.

HRTEM was used to study morphological changes of the nano-precipitates in the nucleation and growth stages and the evolution mechanism of the crystal structure. There were two forms of crystal structure evolution sequence of precipitation: ① B2→Multi-twinned 9R→Detwinned 9R→FCT→FCC and ② B2→Multi-twinned 9R→Detwinned 9R→3R→FCT→FCC (Figure 11). As the nano-precipitate grew, the morphology changed: spherical→ellipsoid→rod-shaped.

In the early nucleation stage of the Cu precipitation phase, Cu atoms aggregated to form a B2 ordered structure, which had superlattice diffraction spots in the reciprocal space. As the B2 ordered structure increased, the BCC lattice could not maintain the stability of the ordered structure. Finally, B2 was transformed into a 9R Cu structure. 9R had a long period characteristic, with each cycle consisting of nine layers, and its close-packed surface spacing was equal to that of FCC Cu. Afterward, 9R Cu underwent a series of martensitic shears, forming a structure containing multiple twin planes, effectively reducing free energy and ensuring stability. 

The volume growth of multi-twin 9R Cu led to an increase in the system’s free energy, forcing the precipitated phase to release the accumulated free energy as much as possible by adjusting its internal lattice structure. The growth of multi-twin 9R Cu resulted in a change in the width of the twin. The movement of the twin interface ultimately promoted the detwinning of 9R Cu and its transformation into the FCT Cu structure. FCT Cu mainly changed the crystal plane spacing and angle through crystal plane rotation, achieving lattice relaxation and ultimately transforming into a stable FCC Cu structure. But the other sequence was different from the above, as 9R Cu underwent twin withdrawing and preferentially transformed into an internal twinned 3R Cu structure. 3R Cu transformed into an FCT structure through lattice plane rotation and changed into crystal plane spacing, ultimately evolving into an FCC Cu structure.

### 3.5. Influence of Processing on Precipitation

Through transmission electron microscopy, the size, quantity, and characteristic distribution of nano-precipitates were analyzed. After different tempering times, the microstructure of the steel did not show significant changes and mainly consisted of tempered bainite. With increased tempering time, the shape of the tempered bainite changed from lath to equiaxed. The average size of the precipitated particles increased, while the quantity density decreased. The yield strength of the steel reached its maximum value of 688 MPa at a tempering time of 1 h. At this point, the density of the precipitated particles slightly decreased compared to 0.5 h, but the number of precipitates increased significantly, with a size distribution mainly in the diameter of 12–26 nm. By analyzing the size statistics and crystallographic sequences of the precipitates, it was concluded that the precipitates mainly consisted of 9R and 3R structures. 

As the tempering time increased to 2 h or more, according to the statistics of precipitated particles, the particle diameter was between 18 and 44 nm, and they mainly consisted of FCT and FCC phases. It can be seen that nano-precipitation, as a precipitation strengthening method, is an effective strengthening method for Cu-bearing ultra-low carbon steel, and a small amount of microalloying elements can significantly improve the strength of steel. In particular, nano-precipitates play an important role in the strengthening process.

## 4. Conclusions

In this study, the crystallographic evolution sequence of nano-precipitation in Cu-bearing ultra-low carbon steel was examined by HRTEM.

(1) There were two crystallographic evolution paths for the morphology and crystal structure changes during the precipitation process: B2→multi twin 9R→detwined 9R→FCT→FCC and B2→multi-twin 9R→detwinned 9R→3R→FCT→FCC. Through the simultaneous existence of 9R, 3R, and FCT structures in the same precipitated particles, it was proven that the evolution sequence of precipitates in Cu-bearing ultra-low carbon steel could have a stable 3R structure.
(2) In addition, the morphology of the precipitated particles during the growth process changed from spherical to ellipsoidal and then to rod-shaped. By observing the nano-precipitates during different heat treatment processes, the structures of B2, 9R, 3R, and FCT were accurately determined.
(3) By analyzing the microstructure and mechanical properties of the steel at different heat treatment processes, it was found that nano-precipitation could effectively improve the yield strength of Cu-bearing ultra-low carbon steel. Statistical analysis of the size and distribution characteristics of precipitation particles revealed that the yield strength of the steel reached its maximum when the precipitation size was mainly 9R structure between 12 and 26 nm.

## Figures and Tables

**Table 1:** Statistical analysis of precipitation particle size distribution and number density under different heat treatment conditions.

Heat Treatment Time/h	Average Size/nm	Number Density/m^−3^
0.5	18.5 ± 3.18	(2.08 ± 0.5) × 10^21^
1	18.6 ± 2.9	(1.78 ± 0.43) × 10^21^
2	28.4 ± 4.8	(1.59 ± 0.39) × 10^21^
2.5	29.2 ± 5.38	(8.36 ± 2.04) × 10^20^

**Table 2:** Mechanical properties of the steel under different heat treatments.

Tempering Time/h	YS/MPa	UTS/MPa	Elongation/%
0.5 h	678	721	22.9
1 h	688	724	24.7
2 h	650	693	24
2.5 h	656	698	25

## Data Availability

All data generated or analyzed during this study are included in this published article.
